# Construing Interpersonal Meaning through Doctors’ Choice of Interrogatives: An Investigation of Doctor-patient Conversations in China

**Published:** 2018-10

**Authors:** Xi LUO, Zhengzhi FENG, Bingjun YANG

**Affiliations:** 1. Dept. of Foreign Languages, School of Basic Medicine, Army Medical University, Chongqing, China; 2. School of Psychology, Army Medical University, Chongqing, China; 3. School of Foreign Languages, Shanghai Jiao Tong University, Shanghai, China

## Dear Editor-in-Chief

Doctor-patient conversations as a special type of institutional conversation carry many interrogatives, embodying information with particular meanings. As to interrogatives from the systemic functional perspective, no study has been found. The purpose of this study was to explore the characteristics of doctor’s choices of interrogatives and how interpersonal meaning was constructed by such choices in doctor-patient conversations in the Chinese context.

On-site real conversations between doctors and out-patients of Southwest Hospital in China were collected in 2014, which involve 11 clinical departments. Altogether 120 conversations were recorded. The raw data were transcribed according to Jefferson’s conversation analytical categories. Overall, 1042 interrogative clauses from doctors were screened out for four different clinical departments, focusing on the types of questioning and moves. Discrepancy was compared with chi-square test. Statistical analysis was conducted using SPSS 19.0 (Chicago, IL, USA).

We found that in Chinese doctor-patient conversations, doctors’ questions occupy about 76.9% of the total questions, about 10% less than the findings (around 86%) from other studies (1–3). Chinese doctors, especially those in the Department of Traditional Chinese Medicine, favored yes-no interrogatives most. Yes-no interrogatives and alternative interrogatives can both be responded to very quickly, and the high frequency of using these interrogatives unconsciously prompts doctors and patients to ignore an effective approach to building harmonious interpersonal relationships during consultation. Good interpersonal relationship may help reduce medical disputes, and may even help improve both doctors’ working environment and patients’ recovering speed. Other factors such as gender, age, and ethnicity are also very important in diagnosis and treatments, and all of which need to be explored. These findings could be used to develop tools for collecting quantitative data on Chinese doctor-patient conversations. Eventually, this study may enhance the efficiency of treatment and decrease medical disputes caused by bad communications.
